# Immersive Virtual Reality Serious Games With DL-Assisted Learning in High-Rise Fire Evacuation on Fire Safety Training and Research

**DOI:** 10.3389/fpsyg.2022.786314

**Published:** 2022-05-23

**Authors:** Shih-Yeh Chen, Wei-Che Chien

**Affiliations:** ^1^Department of Computer Science and Information Engineering, National Taitung University, Taitung, Taiwan; ^2^Department of Computer Science and Information Engineering, National Dong Hwa University, Shoufeng, Taiwan

**Keywords:** serious games, virtual reality, fire safety training, DL-assisted learning, behavioral skills training

## Abstract

In case of fires in high-rise buildings, appropriate safe behaviors for leaving the high floors are the key to reducing injuries and increasing the chance of survival. Traditional training methods are often used to provide knowledge about a fire escape but may become ineffective in terms of knowledge acquisition and internalization. Serious games are an innovative teaching method, aiming at training and educating people in game environments. In recent years, immersive virtual reality has become popular in many educational environments. Various educational training programs are combined with serious games and attract more and more attention because they can make users feel highly involved and promote learning cognition. Therefore, this study proposed the fire safety training of high-rise building fire escape based on virtual reality and invited 140 college students to make explorations through this virtual situation. In addition, deep learning was integrated into the recommended safety training system, so that students could be trained in areas where concepts were ill-defined. According to the results, through the high-rise building fire escape training based on virtual reality, students’ fire safety skills were significantly improved and most students could use their behavioral skills in real situations, which has positive effects on promoting the development of fire escape knowledge. Finally, according to the analysis on the results of the DL-assisted learning system, some suggestions were made in this study on behavioral skills training for professional firefighters and researchers.

## Introduction

Emergency evacuation is very important in fire safety research. According to the investigation on fire injury, if evacuation can be carried out immediately after fires, casualties can be greatly reduced even if fires are not put out quickly. In addition to improper building structures, the main reason for failure to evacuate people from burning buildings in time is that evacuees made unreasonable evacuation choices due to panic or unfamiliarity with buildings (Yang et al., 2003; Lo et al., 2004). Nafiseh et al. (2021) simulated a 28-story multi-purpose high-rise building in their study and predicted the development of smoke and fire on each floor in case of fires. According to the results from fire and smoke simulations and 3D building modeling, it only takes about 400–600 s for smoke and toxic gases to fill all floors of a high-rise building. Even if the building is well designed for fire prevention, there is no guarantee that the requirements of the evacuation plan can be met. In emergency circumstances, appropriate evacuation responses and behaviors are key factors in increasing the chance for survival. People are often trained and educated about an evacuation by traditional methods such as films, posters, seminars, courses, or evacuation drills. However, these traditional methods may not be effective in spreading knowledge (Gwynne et al., 2016), because humans’ psychological reactions and physical behaviors in fires are often very complex and hard to be predicted correctly. When evacuees directly confront life-threatening situations in fire accidents, the increased stress arising from physical threats of fires often affects evacuees’ escape behaviors (Rui et al., 2021). For example, the spread of flames and smoke may cause panic and abnormal behaviors, but building decorations and lighting sometimes may mislead evacuees into choosing the wrong evacuation routes. Therefore, in order to predict human behaviors more accurately, emergency evacuation training and drills in real buildings are effective in perception. However, drills have the disadvantages of high costs, limited repeatability, and dangerousness, and evacuees are not usually provided with individual evacuation behavior assessments after evacuation drills (Gwynne et al., 2017). However, due to the rapid development of virtual reality technology, learners, by combining VR and serious games, can immerse themselves in a virtual building environment with a virtual fire scene to interact with the virtual environment and simulate the process of an emergency evacuation, which overcomes the disadvantages of actual exercises, such as high cost, limited repeatability, and dangerousness. In this case, effective learning environments should be designed, so that learners can practice the measures to take in dangerous situations. However, the immersive design is often insufficient in the virtual environment construction, so that learners cannot immerse themselves in the learning experience when experiencing evacuation, resulting in limited changes in learning attitudes and behavioral responses (Chittaro et al., 2014).

With the improvement of global building technology and limited urban space, high-rise buildings have become common in metropolitan areas. No matter they are used as a residence, commercial offices, or entertainment places, the special building structure and dense population will cause chimney effects in case of fires, resulting in rapid burning, high temperature, dense smoke, and difficulty in escape and rescue. Now, in the context of environmental changes and frequent disasters, everyone should have the basic literacy of disaster prevention, namely knowledge, skills, and attitude, and enrich them, so as to achieve the purpose that prevention is more important than cure (UNESCO, 2005). In recent years, serious games have attracted extensive attention in pedagogy research (Connolly et al., 2012). Serious games are a form of digital games, with the main purpose of training and education rather than entertainment (Connolly et al., 2009). It has been pointed out that, participants can acquire and retain knowledge more effectively by playing serious games than by using traditional learning methods (Wouters et al., 2013), and immersive virtual reality can increase people’s involvement and perception in serious games (Lovreglio et al., 2017). The study on the immersive environment is conducted in many fields but has been the most sufficient in education by far (Suh and Prophet, 2018). It can help participants retain knowledge for longer than traditional learning methods because participants can benefit from full involvement and high levels of emotional and physical arousal (Chittaro and Buttussi, 2015). Therefore, this study proposes and achieves a learning environment based on immersive virtual reality serious games for training behavioral skills on fire safety for high-rise building fire escape. Moreover, the deep reinforcement learning is imported into the level recommendation system for behavioral skills on fire safety, which can adjust the difficulty level and sequence of levels according to participants’ learning status and focus on training participants’ weak skills, so as to improve participants’ learning effectiveness and discuss the following issues:

1. How does the behavioral skills training of the Immersive Virtual Reality Serious Games with DL-Assisted Learning affect the development of behavioral skills for fire safety?
2. How can learners transfer their behavioral skills to real life through the Immersive Virtual Reality Serious Games with DL-Assisted Learning?

The rest of this paper is organized as follows. In section “Literature Review,” we introduce the state-of-the-art literature on virtual reality, fire safety training, and deep learning in education. Then, the research method is shown in section “Research Method.” Section “Experimental Analysis and Discussion” presents the experimental analysis and discussion. Section “conclusion” provides conclusion.

## Literature Review

### Virtual Reality

Virtual reality is to simulate the real world with computer-generated 3D virtual environments, and users can interact with the simulated environment through special wearable devices (Negut et al., 2016). Through head-mounted displays, users will be teleported to virtual environments to become specific virtual characters, and the position information of the head movement will be directed. With the rapid development of virtual reality technology, learners’ perception, presence, and involvement are increased and get close to the reality. In this case, learners feel as if they are part of the reality. The technology can be applied to a wide range of fields because it is flexible and can adapt to different situations. Once participants are immersed in virtual environments, they can feel that they are actually in the computer-simulated environments. Such virtual environments may become very real, making it difficult for individuals to distinguish between virtual and real worlds (LaValle, 2017). Hence, immersive virtual reality has the potential to make participants’ behave and react as close to reality as possible (Sherman and Craig, 2003).

As an ideal tool for exploration, training, and education (Psotka, 1995), immersive virtual reality can be more effective in emergency management training because it can simulate high-fidelity human behaviors (Shendarkar et al., 2008). Compared with non-immersive virtual reality, immersive virtual reality can provide good abilities to memorize and recall, enabling participants to focus on tasks due to a good immersive experience. Moreover, most participants consider that immersive virtual reality can enhance their concept of space and is one of the important factors affecting success experience (Krokos et al., 2018). In addition, immersive virtual reality experience can enhance the educational purposes of serious games, thereby making learning and behavioral outcomes more significantly and involvement higher through the particular cognitive processes (Gao et al., 2017; Lovreglio et al., 2017), and immersing learners in virtual environments, so that they can explore independently, know how to take actions and solve various problems effectively in different situations, start again even if they fail, and can promote the acquire more abilities by adjusting their responses (Buche et al., 2006). Compared with trainings in real environments, the advantages of trainings based on immersive virtual reality technology are safe operation, cost reduction, strong adaptability of situations, personalization settings of teaching suggestions according to training objectives and learners, and flexible adjustment according to the learning experience (Lourdeaux, 2001). Therefore, in some cases, it is very difficult to carry on practical trainings, such as, severe aircraft emergency experience to improve knowledge retention and psychological arousal in aviation safety trainings (Chittaro and Buttussi, 2015), or fire escape. If evacuation drills in the real world, such as heavy smoke or falling objects, are too dangerous to carry out (Kinateder et al., 2014), the combination of immersive virtual reality and serious games will be the best solution, so that participants can understand the dangers and risk awareness.

### Fire Safety Training

Behavioral skills training is used to teach various skills in various situations, including behavioral skills and social skills. As suggested by behavioral skills training, trainees should stay active in learning environments and achieve mastery learning in the teaching process of behavioral skills through the structured model of explanation, demonstration, drill, and feedback (Himle and Miltenberger, 2004). Behavioral skills training is one of the effective methods to obtain natural responses to corresponding situational behaviors in case of an emergency. It can be used to teach personal safety skills to learners of all ages, so as to improve skills in kidnapping prevention, gun safety, sexual harassment prevention, wildlife injury prevention, and fire safety (Houvouras and Harvey, 2014). In skills trainings, trainees interact more actively in simulated environments, try to make mistakes in a comfortable way in dangerous situations, and modify learning experiences (Himle and Miltenberger, 2004). In this respect, it is important to create environments close to reality in behavioral skills trainings, and the lack of the sense of reality will lead to some disadvantages of these environments (Park et al., 2011). However, it is very difficult to create realistic learning environments for trainings about dangerous situations such as earthquakes (Li et al., 2017), floods (Toshio and Kodai, 2020), storms (Henriette et al., 2019), and fires (Ünal and Seyfullah, 2019). In the beginning, the use of virtual reality in learning environments is limited by emulators and high equipment costs. Now, by integrating immersive virtual reality into the Second Life, learning and training activities can be achieved in an interactive, real and secure manner. If virtual reality is used for educational purposes, learning will become fun in interactive, immersive, and engaging environments that are dangerous and impossible to be implemented and experienced in real life (Cha et al., 2012).

The case study on rescue in a smoke-filled building shows that, by comparing participants’ training effectiveness in the virtual reality training at the rescue site and in the actual construction training, participants trained in the real building perform better than those who participate in similar tasks in virtual reality (Bliss et al., 1997). Fire safety trainings in real situations are considered to be the most effective but are expensive and time-consuming (Vanselow, 2013; Buttussi and Chittaro, 2017). In most cases, it is impossible to carry out simulations in trainees’ daily working environments, because activities cannot be interrupted and unpredictable dangers may be caused. Therefore, it is necessary to transfer trainees to dedicated and fully equipped fields. Another limitation is the potential danger of such trainings, which means that fires must always be under control. In this way, the possibility of fires is limited. For safety reasons, these simulated flames always burn in oil or gas tanks, without considering the actual cause of fires. As fires may actually be caused by fuels, chemical gases, and short circuits, the temperature, colors, and smoke of flame all have special characteristics that cannot be fully experienced by trainees in actual trainings. In addition, the fire extinguishing agents used in actual trainings are polluting and their use is limited by environmental regulations. The early virtual reality technology is limited by hardware development. Now, the efficiency of computers and the immersion of head-mounted displays have been greatly improved, indicating that the immersive virtual reality has become a method to overcome these limitations. However, as we know, training in a virtual environment has gradually become the future trend. It can improve learners’ motivation and involvement, help learners to acquire knowledge more easily than the traditional teaching process (Chittaro and Ranon, 2007), make the dangerous teaching process more realistic and safer (Johnson and Levine, 2008; Freina and Ott, 2015), and has important potentials for fire safety trainings. Due to the changes in lifestyle, this study focuses on the high-rise building fires that modern people are prone to encounter and examines its contributions to fire safety skills during training.

### Deep Learning in Education

Effectively instructing students in the learning process is a recurring challenge in current educational research (Minn, 2022). Whether students are trained by teachers or by an AI-assisted system, the assessment of knowledge is necessary to measure their learning outcomes, understand their learning and analyze learning effectiveness. The assessed information can be used to provide immediate feedback (Shute, 2008) and recommend personalized learning materials based on corresponding strategies (Fu and Zhang, 2013). Some research has shown that providing knowledge assessments during students’ learning can improve their motivation and learning efficiency (Bennett, 2011; Moss and Brookhart, 2019). Deep learning is a subcategory of artificial intelligence and is seen as one of the channels for providing precision education (García et al., 2007; Tsai et al., 2020). Since deep learning has the ability to develop and imitate human decision-making processes (Baradwaj and Pal, 2012) and build automated and accurate teaching and learning models, it has played a great auxiliary role in the teaching methods of adaptive education (Colchester et al., 2017). Many teachings have gradually introduced AI/DP to further assist students’ learning. Naik and Ragothaman (2004) proposed a system for predicting the performance of MBA students prior to enrolling at a particular institution. The results show that the proposed method is far superior to other statistical processes in terms of reliability and commitment. Ibrahim and Rusli (2007) and Idris et al. (2009) adopt neural networks to predict students’ academic performance and identify learning objectives. In addition, the result shows that ANNs can also be used to find out which learning objects are most suitable for a particular person. Beetham and Sharpe (2013) introduced the work of learning objects for classification based on the concept of artificial neural network (ANN). The authors employ backpropagation (BP) and self-organizing map (SOM) algorithms to establish the link between the domain concepts of the learning objects and the learning needs of the learners to provide learning objects that are suitable for each user. The results also show that teacher duties and rule-based choices can be replicated by adaptive models created using ANNs. Chocarro et al. (2021) study teachers’ acceptance of AI chatbots. The result shows that ease of use and utility plays a key role in accepting artificial intelligence.

## Research Methods

Inspired by the functions and potentials of immersive virtual reality, this study focuses on combining it with serious games and introducing DL-assisted learning technology, in order to provide information about correct evacuation in case of high-rise building fires. Due to the difficulties and potential dangers of conducting trainings in actual situations, virtual reality technology in such situations is considered a major advance as it can reduce training costs and provide safe learning environments close to reality. Most studies focus on the link between immersion and presence show that hardware that provides strong immersion provides good presence, and vice versa. However, this learning method is often the conceptual learning which cannot provide the procedural learning of actual fire extinguisher operation and fire hose laying to put out fires. Therefore, in this study, a training situation combining virtual high-rise building fire escape and real high-rise building fire drill is proposed to explore whether the interaction between conceptual learning and procedural learning can help transfer behavioral skills to real life, and it is expected that a conceptual framework will be developed based on the implementation standards and expert opinions of fire safety drills provided for system development by professional firefighters as the major achievement of this study.

### Research Design and Process

In Step 1, trainees are required to complete questionnaires to assess various factors, including participants’ gender, age, gaming experience, computer experience, and fire safety experience, as well as questionnaires to assess skills and abilities. The pre-testing of fire safety knowledge should be conducted prior to the instruction of training activities, and the test is constructed with the assistance of fire protection professionals according to the teaching objectives of the training in this study. After listening to the activity instruction, participants are guided to take the training course, including procedural training with practical drills and conceptual training with virtual reality. First, an actual high-rise building fire escape drill is carried out, and participants listen to the operation instructions on immersive virtual reality serious games after a short rest, then high-rise building fire escape is simulated in the virtual reality. After the above training, each trainee is given 30 min to complete the knowledge post-test and presence questionnaire, and have a qualitative interview with the researcher.

### Experimental Pre-test and Post-test

Our study assesses two learning types, namely conceptual knowledge test and procedural skill test. An experimental pre-test is conducted prior to the high-rise building fire safety training, and trainees must complete questionnaires to assess their initial level of knowledge and skills in fire safety. The conceptual knowledge test contains 10 items, with a full score of 50 points. These questions are related to theories and knowledge required in high-rise building fires, and the initial state of their knowledge is tested according to the educational objectives of the safety training, such as do you understand fire risks, do you know the chimney effect, and which fire safety equipment do you know. In the procedural skill test, professional firefighters provide pre-test situations to participants in the form of role play before the training course, to access their initial state of procedural skills in the field according to their responses to and operations in fires, such as small fires in high-rise building staircases, big fires in high-rise building staircases or escape routes blocked by big fires. The evaluation factors are the relevance and coherence of information collected by learners in the case of high-rise building fire escape, as well as decision execution order and method. Professional firefighters will score them, with the full score of 50 points and a total of 100 points.

A post-test is conducted after the high-rise building fire safety training, including conceptual knowledge test and procedural skill test as same as the pre-test. Similar to the pre-test, the conceptual knowledge post-test contains 10 items, with a full score of 50 points. Trainees must complete questionnaires. During the interviews with fire protection professionals and learners and the fire safety training, fire protection professionals verify their knowledge acquisition by observing trainees’ responses to assess their knowledge development. The procedural skill test, the post-test similar to pre-test in the form of role play, is after the training course. When the fire cannot be put out, participants’ skill development is observed and scored with the full score of 50 points and a total of 100 points.

### Trainings Through Actual Drills and Based on Immersive Virtual Reality Serious Games With DL-Assisted Learning

As a part of this study, we propose a training of high-rise building fire safety. A 10-story building is used as the training situation. How should trainees respond when a fire breaks out? Escaping to a safe assembly point will be the focus of this training. In this training, actual drills are conducted to acquire procedural skills, such as laying fire hoses and using copper fire nozzles to put off fires. This skill is dangerous in operation because it is easy to hit yourself or others with wrong skills in throwing and retracting fire hoses, which cannot be experienced in virtual reality. In addition, it is difficult to set a real fire source in the high-rise building fire simulation, due to the chimney effect. Slight carelessness can easily lead to unexpected accidents. Therefore, virtual fire sources are always set, which makes trainees taken them lightly, without the feeling of immersion. Therefore, after the actual drill, it is necessary to carry out the training based on immersive virtual reality serious games with DL-assisted learning, which improves their presence in the fire.

A total of 10 situations and 5 levels, each level has two situations, are designed for the drill, and the physical drill is as same as the virtual drill. Trainees are led to their rooms in the beginning and asked to get to a safe assembly point as quickly as possible when smoke fills the high-rise residence. The training content has been verified by fire training professionals, including some wrong responses that mislead trainees, so as to judge their knowledge internalization. For example, it is considered necessary to cover your nose and mouth with a wet towel when trying to escape a fire, which is wrong in fact, because the vapor generated when the wet towel is heated may cause serious burns if inhaled into the respiratory tract. You can wear a firefighter breathing apparatus, if not, try to leave the fire site within the shortest period.

**FIGURE 1:**
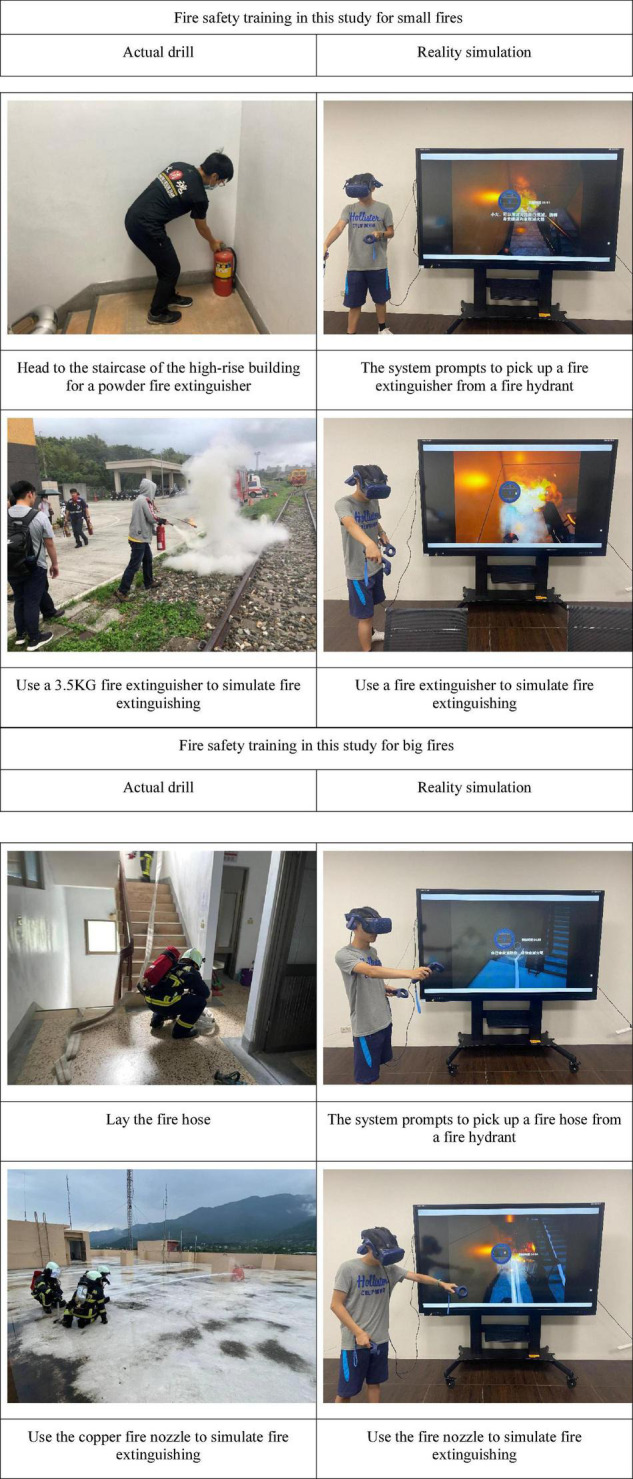
Fire safety training in this study (actual drill and reality simulation).

It is recommended that the trainees take flashlights. If they are not familiar with the building layout, they can look at the directions guided by the emergency escape signs on the escape plan and walk downstairs by the escape ladder. When seeing the alarm bell, he should press it to start the fire service installation. When reaching the fifth floor, he will encounter a small fire as shown in Figure 1. After that, the trainee should return to the fire hydrant on that floor, take the fire extinguisher to extinguish the fire, and then continue to escape. When reaching the second floor, he will encounter a big fire as shown in Figure 1. At this time, the trainee can choose to return to the fire hydrant on that floor, take the fire hose and fire nozzle to extinguish the fire. If the fire is too big, he can choose to return to the room, wet the bath towel to stuff the gap of the door, pour water over the door with a basin to cool it down, and find a safe rappelling tool, or choose other self-rescue models.

In each level, virtual reality serious games will give 5–10 hints according to the difficulty level of each level. Each participant needs to participate in 2 rounds of the game. The prompts in the first round are complete, and all hints will be given in the first round. In the second round, the ANN model will be used to estimate the number of hints at each level for each participant. In data preprocessing, we label the data with the help of experts. The features of these data are captured by continuously training ANN models. We convert this problem into a classification problem and adopt the ANN model to find the appropriate number of hints for participants in each fire safety training level according to the user’s reaction time, the correctness of operation, and the smoothness of operation. If the participant is very unfamiliar with the fire safety training process, it is still possible to get all the hints in the second round. However, for the more proficient users, no hints may be given. Virtual reality serious games without hints can enhance the gaming experience and further verify the effectiveness of fire safety training.

Finally, the presence is assessed with the Igroup Presence Questionnaire (IPQ) published by Schubert et al. (2001). Over the years, with the advance in virtual reality, various questionnaires for presence assessment have been developed, and the definition of presence has been constantly updated. Some people assess the presence, while others are interested in different dimensions of the presence. We choose IPQ because it has been widely used in similar studies.

## Experimental Analysis and Discussion

In order to understand the relationship between the immersive virtual reality serious games with DL-assisted learning and high-rise building fire safety skills, subjects are not limited to those with fire experience. In this study, a total of 156 questionnaires were issued to the junior students of the Institute of Science and Technology at the National Taitung University. After the incomplete questionnaires were deducted, there were a total of 140 questionnaires. SPSS statistical software was used to process the results. A one-way analysis of variance was firstly carried out, including 112 males and 28 females, with an average age of 21.02, standard deviation of 1.10 [*F*(3.39) = 0.53, *p* > 0.5], computer experience of [*F* (3.39) = 0.56, *p* > 0.5], gaming experience of [*F*(3.39) = 0.19, *p* > 0.5], expertise in fire safety of [*F*(3.39) = 0.34, *p* > 0.5], to confirm no significant differences between subjects.

In order to verify whether the IPQ scale used in this study was appropriate for the collected data, an exploratory factor analysis (EFA) was firstly conducted on the scale for necessary revision. In the exploratory factor analysis, the princip1e component ana1ysis was adopted, then the factors with eigenvalue greater than 1 were extracted by the varimax orthogonal rotations, and the questions with the factor loading lower than 0.40 were omitted. According to the results (as shown in Table 1), Question 11 was deleted for its low factor loading (< 0.40). EFA was carried out again for the remaining 12 questions. After the exploratory analysis, orthogonal rotations were abandoned and oblique rotations were adopted. After comparison, the unweighted least square method and EFA with oblique rotations were used to give a 3-factor pattern, with the total variance explained of 58.03%, to rotate to a 3-factor pattern. Projects IPQ2-IPQ6 indicated the spatial presence (SP), projects IPQ7-IPQ10 indicated the involvement (INV), and projects IPQ12-IPQ14 indicated the reality (REAL). In terms of reliability, Cronbach’s α was used to test the internal consistency. According to the results, Cronbach’s α was 0.84 for the spatial presence, 0.79 for the involvement, 0.76 for the reality, and 0.87 for the whole scale, indicating that this scale has acceptable reliability and validity.

**TABLE 1:** Factor analysis results of IPQ.

Item	SP	INV	REAL
IPQ2	0.800		
IPQ3	0.734		
IPQ4	0.712		
IPQ5	0.683		
IPQ6	0.679		
IPQ7		0.715	
IPQ8		0.675	
IPQ9		0.662	
IPQ10		0.652	
IPQ12			0.741
IPQ13			0.725
IPQ14			0.658
Characteristic value	4.39	3.14	2.92
Explained variation	24.38%	17.43%	16.23%
Total variance explained		58.03%	

And then, the effects of immersive virtual reality serious games with DL-assisted learning (presence, involvement, and reality) on high-rise building fire safety skills are explored. Hence, multiple stepwise regression is used to explore which variables can effectively predict knowledge about fire safety skills. Table 2 shows the multiple stepwise regression analysis on the knowledge about high-rise building fire safety skills based on immersive virtual reality serious games. According to the changes of the determination coefficients in Table 2, when there is the only predictive variable of presence, 38% variance of the knowledge about fire safety skills can be explained (F1,382 = 230.06,*p* < 0.05); after the explanatory power of presence is excluded, the addition of involvement can increase the variance explained by 9% [*F*(1, 382) = 67.90, *p* < 0.05]; after presence and involvement are excluded, the addition of reality can increase the variance explained by 1% [*F*(1, 382) = 6.16, *p* < 0.05]; the accumulated determination coefficient of the three predictive variables is.48, reaching a significant level [*F*(3, 380) = 116.33, *p* < 0.05] and indicating that the combination of presence, involvement and reality can explain 48% variance of the knowledge about fire safety skills. The normalized regression equation is “knowledge about fire safety skills = 0.36* presence + 0.32* involvement + 0.12* reality.” The positive and negative β values show that presence, involvement, and reality are proportional to the knowledge about fire safety skills, indicating that more knowledge about fire safety skills can be obtained with stronger presence, involvement, and reality.

**TABLE 2:** Prediction of fire safety knowledge and fire fighting skills.

Independent variable	Raw-score regression coefficient	Standardized regression coefficients	Coefficient of determination (*R*^2^)	Coefficient of determination cumulative	*F*
SP	1.65	0.36	0.38	0.38	116.33*
INV	0.87	0.32	0.09	0.47	
REAL	0.46	0.12	0.01	0.48	
Interpolation	10.59				

*p < 0.05 (significant), **p < 0.01 (highly significant); ***p < 0.001 (extremely significant).

According to the above results, presence and involvement have good predictive power for personal knowledge about fire safety skills, and the predictive power of reality is only 1%. Researchers suggested that the reality should have quite good effects on high-rise building fire experience theoretically, but not everyone has fire experience and one is unable to experience the reality brought by high temperature and choking smoke at fire sites without actual experience. Hence, in this study, the subjects paid less attention to the knowledge about fire safety skills acquired by the dependent variable of reality in high-rise building fires.

According to the pre-test and post-test of fire safety knowledge before and after training, the paired-samples *t*-test is analyzed, as shown in Table 3. According to the table, the t value of the paired-samples test is -5.41 and reaches the significant level of 0.05, indicating that there is a significant difference in the knowledge about fire safety skills acquired from the training based on immersive virtual reality serious games, but trainees’ performance after self-directed training (*M* = 82.10) is significantly better than that before training (*M* = 77.30).

**TABLE 3:** Paired sample *t*-test between pre-test and post-test scores of fire safety knowledge.

Item	M	N	SD	T
Pre-test	77.30	140	6.70	−5.41***
Post-test	82.10	140	6.08	

*p < 0.05 (significant), **p < 0.01 (highly significant); ***p < 0.001 (extremely significant).

In the experiments, the qualitative data, such as high-rise building fire training based on immersive virtual reality serious games, fire protection professionals, and some trainees’ interviews, are used to understand the effects of the system on learning outcomes of applying fire safety skills to high-rise building fires for a conclusion. Without warning, about half of the trainees are found not to press the alarm bell before performing evacuation. Their feedback is that it is easy to overlook these small things in tense situations. Moreover, about a third of the trainees, affected by stereotypes, will choose to escape from the top floor in case of high-rise building fires. The move often leaves them trapped due to the chimney effect and missing the golden opportunity for escape. In general, most trainees give positive feedback and agree that they obtain different high-rise building fire escape experience. However, a small number of trainees suffer from dizziness during the virtual reality serious game experience due to nervousness but are relieved from discomforts after slight adjustments.

## Conclusion

This study employed the immersive virtual reality serious games with DL-assisted learning to conduct fire safety training for dangers in real life. In the behavioral skills training conducted through virtual reality serious games, the immersion quality of games and the presence of participants are the key factors affecting the development of fire safety skills. In this aspect, virtual reality serious games should be developed and designed in a more realistic and immersive way. In order to avoid dizziness, appropriate sound and vibration can effectively reduce the discomforts that can easily occur in the virtual reality experience and improve control stability (Sawada et al., 2020). On the other hand, when fire safety training situations are designed for virtual reality serious games, it is important to design the right behavioral skills for game scripts. According to studies, people’s behaviors can be accurately highlighted when immersive virtual reality serious games are combined with actual on-site training. Studies have pointed out that skills, behaviors, and learning outcomes acquired in virtual reality environments can still be recalled after 3 months (Grabowski and Jankowski, 2015). Therefore, immersive virtual reality serious games can be used as an alternative for fire safety training, which can achieve an experience perception close to the reality in highly immersive serious games. We hope the results will contribute to the design and development of future learning environments based on virtual reality technology, as well as the use of fire safety training in the immersive virtual reality serious games with DL-assisted learning. However, the limitation of this research is that this game cannot achieve multi-user connections. Therefore, it cannot meet the requirements of multiple user training at the same time and cultivate the ability of teamwork. In future studies, a distributed interactive simulation will be developed and allow multiple users to participate in the evacuation at the same time.

## Data Availability Statement

The raw data supporting the conclusions of this article will be made available by the authors, without undue reservation.

## Ethics Statement

Written informed consent was obtained from the individual(s) for the publication of any potentially identifiable images or data included in this article.

## Author Contributions

W-CC contributed to conception and design of the study. S-YC organized the database and performed the statistical analysis. Both authors contributed to manuscript revision, read, and approved the submitted version.

## Conflict of Interest

The authors declare that the research was conducted in the absence of any commercial or financial relationships that could be construed as a potential conflict of interest.

## Publisher’s Note

All claims expressed in this article are solely those of the authors and do not necessarily represent those of their affiliated organizations, or those of the publisher, the editors and the reviewers. Any product that may be evaluated in this article, or claim that may be made by its manufacturer, is not guaranteed or endorsed by the publisher.
